# A Neurodevelopmental Model of Combined Pyrethroid and Chronic Stress Exposure

**DOI:** 10.3390/toxics7020024

**Published:** 2019-05-02

**Authors:** Aimée I. Vester, Merry Chen, Carmen J. Marsit, W. Michael Caudle

**Affiliations:** 1 Department of Environmental Health Sciences, Emory University Rollins School of Public Health, Atlanta, GA 30329, USA; aimee.vester@emory.edu (A.I.V.); merry.chen@emory.edu (M.C.); carmen.j.marsit@emory.edu (C.J.M.); 2 Center for Neurodegenerative Disease, Emory University, Atlanta, GA 30322, USA

**Keywords:** corticosterone, deltamethrin, dopamine, neurodevelopment, pyrethroid

## Abstract

Attention-deficit hyperactivity disorder (ADHD) is one of the most common neurodevelopmental disorders of childhood and previous studies indicate the dopamine system plays a major role in ADHD pathogenesis. Two environmental exposures independently associated with dopaminergic dysfunction and ADHD risk include exposure to deltamethrin, a pyrethroid insecticide, and chronic stress. We hypothesized that combined neurodevelopmental exposure to both deltamethrin and corticosterone (CORT), the major stress hormone in rodents, would result in additive changes within the dopamine system. To study this, we developed a novel dual exposure paradigm and exposed pregnant C57BL/6 dams to 3 mg/kg deltamethrin through gestation and weaning, and their offspring to 25 μg/mL CORT dissolved in the drinking water through adulthood. Midbrain RNA expression as well as striatal and cortical protein expression of key dopaminergic components were investigated, in addition to ADHD-like behavioral tasks and electrochemical dopamine dynamics via fast-scan cyclic voltammetry. Given the well-described sexual dimorphism of ADHD, males and females were assessed separately. Males exposed to deltamethrin had significantly decreased midbrain *Pitx3* expression, decreased cortical tyrosine hydroxylase (TH) expression, increased activity in the Y maze, and increased dopamine uptake rate in the dorsal striatum. These effects did not occur in males exposed to CORT only, or in males exposed to both deltamethrin and CORT, suggesting that CORT may attenuate these effects. Additionally, deltamethrin- and CORT-exposed females did not display these dopaminergic features, which indicates these changes are sex-specific. Our results show dopaminergic changes from the RNA through the functional level. Moreover, these data illustrate the importance of testing multiple environmental exposures together to better understand how combined exposures that occur in certain vulnerable populations could affect similar neurodevelopmental systems, as well as the importance of studying sex differences of these alterations.

## 1. Introduction

Attention-deficit hyperactivity disorder (ADHD) is one of the most common neurodevelopmental disorders of childhood [1]. Characteristics include symptoms within three domains: inattention, hyperactivity, and impulsivity, that manifest by age 12 and can persist into adulthood [2,3]. To date, no singular pathogenic mechanism of ADHD is known, and ADHD is likely to be multifactorial, involving genetic, epigenetic, and environmental components. Several monoaminergic neurotransmitter circuits have been implicated in ADHD. Notably, genetic studies reveal variants in several genes related to the dopamine system are associated with ADHD, including dopaminergic receptors, enzymes, and transporters [4,5,6,7]. Variants in the dopamine receptor 5 gene (*DRD5*) modulate age of ADHD onset, while variants in the dopamine transporter (*DAT1*) gene predict severity of hyperactivity and impulsivity symptoms [8,9]. Lower DNA methylation of *DRD4* is also associated with an increase in ADHD symptoms in children at age 6 [10]. Cohort studies associate altered DAT levels with ADHD [11,12,13] and functional MRI studies indicate that dopaminergic brain regions are important in children with ADHD and ADHD-associated SNPs (single nucleotide polymorphisms) in the *DAT1* gene [14,15]. Additionally, a *Dat1* knockout mouse displays hyperactivity, while a *Dat1* overexpressing mouse model shows impulsivity behaviors [16,17,18,19,20]. Finally, methylphenidate targets the dopamine and norepinephrine reuptake inhibitors and a differential drug response is associated with *Dat1* genotype [21]. Based on these genetic, mechanistic, and pharmacologic data, there is strong evidence for a role of dopaminergic signaling in ADHD pathogenesis. 

Dopamine is a catecholamine neurotransmitter synthesized by tyrosine hydroxylase (TH) and metabolized by catechol-O-methyltransferase (COMT) and monoamine oxidase (MAO) enzymes [22], and subsequently transported into dopaminergic vesicles by the vesicular monoamine transporter 2 (VMAT2) [23]. Upon stimulation, vesicles release dopamine across the synapse [24]. Dopamine interacts with postsynaptic dopamine receptors (DRD1–5) and is then recycled via DAT or metabolized after neurotransmission [24]. Dopamine is primarily produced by dopaminergic cell bodies in the midbrain, specifically in the substantia nigra pars compacta (SNpc) and ventral tegmental area (VTA) [25]. The SNpc sends projections to the dorsal striatum, where dopaminergic signaling plays a key role in learning and motor function [26]. The VTA sends projections to the nucleus accumbens, olfactory bulb, amygdala, hippocampus, prefrontal cortex, and cingulate cortex. In these areas, dopamine modulates motivation, reward-related behavior, attention, and memory [26]. Many of these dopaminergic networks are implicated in ADHD [27].

While extensive genetic studies suggest that ADHD is familial, this does not preclude exogenous risk factors that further modulate risk of the disease [28]. Studies of various perinatal and postnatal environmental exposures including maternal smoking [29], early childhood adversity [30,31,32,33], and exposure to environmental toxicants have been linked with ADHD [28,34,35]. The neurodevelopmental process is especially vulnerable to environmental disruption because environmental contaminants may cross the placental barrier, pass through the immature blood-brain barrier, or transfer into breast milk where additional exposure can occur [36,37,38,39]. ADHD prevalence is higher in low-income children [40], suggesting environmental risk factors affect certain socioeconomic groups differentially and contribute to this health disparity. Two environmental risk factors linked to ADHD and low socioeconomic status are exposure to pyrethroid insecticides and chronic psychosocial stress [30,41,42,43,44]. 

Pyrethroid insecticides are synthetic analogues of pyrethrins, compounds derived from *Chrysanthemum cinerariaefolium*, that bind the α subunit of voltage-gated Na^+^ channels and hold them open to cause neuronal hyperexcitability [45]. Type II pyrethroids, such as deltamethrin, can also inhibit GABA_A_ receptors and voltage-gated chloride channels to potentiate neuronal excitability [46]. Although pyrethroids are known to target neuronal channels present in most neurons, work from our group and others has shown that the dopamine system is uniquely vulnerable to pyrethroid exposure. Treatment of adult mice with pyrethroids increases maximal dopamine uptake and increases striatal DAT levels [47,48]. This increase in dopamine uptake is mediated by DAT [49], and alterations in dopamine release were replicated in experiments directly injecting pyrethroids into the rat striatum [50]. More recently, we discovered that exposure to deltamethrin during critical periods of neurodevelopment results in similar alterations in the dopamine system. Specifically, we observed increased DAT and dopamine receptor 1 (DRD1) expression that was associated with hyperactivity and impulsivity, as well as deficient memory and attention behaviors that reflect clinical symptoms of ADHD [41]. In addition to animal studies, elevated levels of pyrethroids are associated with ADHD in humans. Notably, children in the National Health and Nutrition Examination Survey (NHANES) that have detectable pyrethroid metabolites in urine have an increased risk of ADHD [41]. This association appears to be higher in boys, and in children with hyperactive-impulsive symptoms [51]. Together, these results indicate that pyrethroids alter dopamine activity, and pyrethroid exposure is an important ADHD risk factor to evaluate. 

Another environmental risk associated with ADHD is socioeconomic status [30,52]. At least part of this risk has been attributed to the higher levels of psychosocial stress experienced by individuals in these communities due to factors within their homes and communities [53,54,55]. This psychosocial stress activates the hypothalamic–pituitary adrenal axis to release the major stress hormone, cortisol in humans and corticosterone in rodents [56]. Studies measuring children’s hair cortisol levels show an inverse relationship between socioeconomic status and cortisol [57,58], suggesting effects of disproportionate psychosocial stress start early in life. In rodent models, corticosterone has been shown to interact with the dopamine system: acute stressors such as a tail pinch induce dopamine release in the medial prefrontal cortex, and this is regulated by glucocorticoid action on dopamine neurons in the VTA [59]. Rodent pups separated from their mothers, a model of early life stress, exhibit hyperactivity and decreased attention that improves with methylphenidate [44]. Additionally, rats subjected to a visible burrow system, a chronic stress paradigm, exhibit decreased DAT and DRD2 expression in the striatum [60]. These results indicate that chronic stress influences dopamine signaling and neurologic alterations that may underlie ADHD. Interestingly, glucocorticoid receptors, which potentiate the signaling of the stress hormone cortisol, co-localize with dopamine neurons in the SNpc and VTA [61], suggesting that stress may impact development and function of the dopamine system. In addition to experiencing greater amounts of psychosocial stress, low socioeconomic status groups are more likely to be exposed to environmental contaminants [62], including pyrethroid insecticides. Elevated levels of pyrethroids have been observed in low-income, urban populations, likely due to poor housing conditions and increased need for pest control [63,64,65]. Certain populations may therefore be exposed to high amounts of both cortisol and pyrethroid insecticides that could modulate risk of dopaminergic dysfunction and development of ADHD.

We hypothesized that combined pyrethroid insecticide and stress hormone exposure would lead to additive changes in RNA expression, protein expression, and function of key components of the dopamine system, thereby potentially increasing the risk of developing ADHD in certain populations. Previous studies have investigated the effects of singular environmental exposures on the development of the dopamine system. However, we developed a novel neurodevelopmental mouse model of combined exposure to deltamethrin, a pyrethroid insecticide, and corticosterone (CORT), the major stress hormone in rodents, from gestation through adulthood. Our exposure approach is the first to describe dopaminergic RNA expression, protein expression, and functional effects in response to these two exposures together, and is also the first to describe sex-specific dopaminergic effects of isolated deltamethrin and oral CORT exposure in greater detail. We illustrate that there are effects of deltamethrin on dopaminergic gene expression, protein expression, behavior, and release kinetics. Interestingly, our results show that these effects are not additive as we initially hypothesized, though our results are sex-specific and contribute to the current understanding of the well-described sexual dimorphism of ADHD. 

## 2. Materials and Methods 

### 2.1. Animals

Eight-week-old C57BL/6NCrl wild-type mice (Charles River Labs, Wilmington, MA, USA) received food and water *ad libitum* and were maintained on a 12:12 dark/light cycle. Females were dosed with 3 mg/kg deltamethrin (Sigma-Aldrich, St. Louis, MO, USA) every three days for two weeks prior to breeding in triplicate. Deltamethrin dosing continued during breeding and gestation. One male and one female mouse per litter were utilized for each experiment to reduce confounding due to litter effects. Throughout, an independent litter represents one sample (*n* = 1). All experiments were approved by the Institutional Animal Care and Use Committee at Emory University (Protocol DAR-4000074-ENTRPR-N, approved at 14 September 2017) and were conducted in accordance with the National Institutes of Health Guide of Care and Use of Laboratory Animals.

### 2.2. Exposure Paradigm

**Figure 1:**
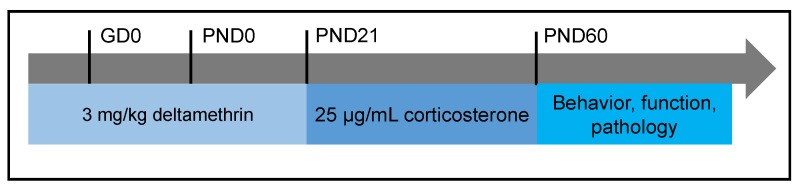
Exposure paradigm timeline. Adult C57BL/6NCrl females were exposed to deltamethrin or vehicle every 3 days during gestation, lactation, and weaning of offspring at postnatal day (PND) 21. Deltamethrin was administered via corn oil dissolved in peanut butter to minimize trauma to pregnant dams. Corticosterone was dissolved in the drinking water to minimize handling stress and reduce variation in CORT levels seen in behavioral chronic stress paradigms.

We utilized a deltamethrin dose of 3 mg/kg based on our previous study showing dopaminergic effects upon neurodevelopmental exposure to 3 mg/kg deltamethrin in male offspring. Additionally, this dose models a realistic exposure in the human population [66,67,68,69,70,71,72,73,74] and is lower than the developmental no observable adverse effect limit (NOAEL). Adult C57BL/6NCrl females were randomized to the deltamethrin or vehicle treatment group and were dosed every 3 days during gestation, lactation, and weaning at postnatal day (PND) 21. Deltamethrin was administered via corn oil dissolved in peanut butter to minimize trauma to pregnant mice. Upon weaning, litters were randomized to receive either oral corticosterone (CORT; Steraloids Inc., Newport, RI, USA) dissolved in drinking water or a drinking water vehicle. CORT was dissolved in the drinking water to minimize handling stress and reduce variation in CORT levels seen in behavioral chronic stress paradigms [75,76]. CORT doses were prepared as previously described [77]. Offspring were continuously exposed to CORT or drinking water vehicle from adolescence through adulthood (PND21–60) (Figure 1). We chose our CORT exposure paradigm because we wanted to model a more persistent state of chronic stress than oft-used behavioral chronic mild stress paradigms [77]. Behavioral chronic mild stress paradigms involve randomization of stressful conditions such as damp bedding, a reversed dark/light cycle, and cage tilting; they are often highly variable in administration and outcomes [76]. Water bottles were weighed daily to assess intake and mice were weighed at weaning and at least once a week to assess proper weight gain. At PND60, offspring either underwent behavioral testing or were sacrificed for fast-scan cyclic voltammetry. All remaining offspring were sacrificed for molecular and pathologic studies. 

### 2.3. Behavioral Analyses

One male and one female offspring from each litter were tested at 8–10 weeks of age. Mice were habituated to the testing room for at least one hour before testing. Behavioral tests were conducted during the same time window for each test by the same set of researchers to reduce confounding due to circadian and olfactory cues. For all animals, the order of behavioral testing was: (1) locomotor activity, (2) Y maze, and (3) marble burying.

#### 2.3.1. Locomotor Activity

To assess locomotor activity, mice were placed in sound-attenuated chambers equipped with photo beams (Med Associates, St. Albans, VT, USA). Mice were habituated for 30 min and then observed for an additional 90 min. Number of ambulations, measured by the number of beam breaks, was quantified in 5-min intervals after the habituation period. 

#### 2.3.2. Y Maze

Mice moved freely in the three-arm maze for eight min total and were recorded with a digital video camera. Noldus EthoVision XT Version 13 software (Wageningen, The Netherlands) tracked alternation behavior and total distance traveled in the maze. One arm entry was scored whenever a mouse entered all four paws into a maze arm. An error consisted of an entry into a maze arm that was the same as one of the previous two arm entries. Percent error was calculated by dividing the number of errors by the total entries – 1. 

#### 2.3.3. Marble Burying

Twenty marbles were placed in a 4 × 5 pattern on top of 2 inches of fresh bedding in a clean cage. Mice were placed at the lower right corner of the cage and allowed to explore freely for 30 min. After 30 min of exploration, mice were removed, and two independent observers scored the total number of marbles buried at least 50%. Any discrepancies greater than 2 marbles were discussed and scores were averaged for each animal. 

### 2.4. Serum Corticosterone Enzyme-Linked Immunsorbent Assay (ELISA)

Following decapitation, trunk blood was collected from mice between 8–10 weeks of age and placed into 1.5 mL centrifuge tubes and allowed to clot at room temperature for one hour. Samples were centrifuged at 10,000 rpm for 10 min at 4 °C. Serum was carefully pipetted off, placed into a clean 1.5 mL centrifuge tube, and stored at −80 °C until ELISA analysis. ELISAs were performed per manufacturer guidelines (Enzo Life Sciences, Farmingdale, NY, USA) and samples were run in triplicate and averaged for each animal. 

### 2.5. mRNA Expression Analysis

Mice underwent rapid decapitation at 8–10 weeks of age and the midbrain was isolated and immediately flash frozen. Total RNA and DNA were extracted with a Qiagen Allprep DNA/RNA Mini Kit. DNA was saved and RNA was extracted per manufacturer recommendations (Qiagen, Germantown, MD, USA). 10 ng total RNA was converted to cDNA using SuperScript IV Reverse Transcriptase (Thermo Fisher Scientific, Waltham, MA, USA) and the PCR cycle conditions recommended by the manufacturer: 94 °C – 2:00 m; 35 cycles: 94 °C – 0:15 m, 55 °C – 0:30 m, 68 °C – 1:00 m; 4 °C – hold. Taqman gene expression assays were utilized for the genes of interest and are listed in Supplemental Table S1. Genes of interest included genes critical for dopamine transport (*Dat1*, *Vmat2*), dopamine synthesis and metabolism (*Comt*, *Th*), transcriptional regulation of dopamine genes (*Nurr1*, *Pitx3*), and the glucocorticoid receptor (*Nr3c1*). Each assay plate contained a non-template control, positive control derived from pooled adult mouse brain tissue, and a beta actin internal standard. ^ΔΔ^Ct values were calculated for each animal at every gene tested, and results are expressed relative to gene expression of the vehicle/vehicle control group.

### 2.6. Immunoblotting

Striatal and cortical samples were dissected upon rapid decapitation of animals at 8–10 weeks of age and immunoblotting was performed as previously described [78]. Striata were homogenized, subjected to PAGE and electrophoretically transferred to polyvinylidene difluoride (PVDF) membranes. Membranes were blocked in 7.5% nonfat dry milk in tris-buffered saline solution to reduce nonspecific binding and incubated overnight in primary antibody. We utilized horseradish peroxidase secondary antibodies (Jackson ImmunoResearch, West Grove, PA, USA) and enhanced chemiluminescence using SuperSignal West Dura Extended Duration Substrate (Thermo Fisher Scientific, Waltham, MA, USA). Luminescence and subsequent densitometric analysis were performed using the ImageLab Version 3.0 software (Bio-Rad, Hercules, CA, USA), and all expression values were normalized to β-actin. Samples across multiple gels were randomized on exposure group and sex to avoid batch effects. Primary antibodies were utilized at the following dilutions: rat anti-DAT (1:1000, Millipore, Burlington, MA, USA), polyclonal rabbit anti-VMAT2 serum (1:5000, Covance Custom Immunology Services, Denver, PA, USA), rabbit anti-TH (1:1000, Millipore, Burlington, MA, USA), mouse anti-COMT (1:1000, Novus Biologicals, Centennial, CO, USA), and mouse anti-β-actin (1:5000, Sigma-Aldrich, St. Louis, MO, USA); all are monoclonal unless otherwise noted. The corresponding secondary HRP-linked antibodies were used (1:7500, Jackson ImmunoResearch, West Grove, PA, USA). The rabbit polyclonal VMAT2 antibody was raised against the C-terminal region of mouse VMAT2 as previously described by our group [79].

### 2.7. Fast-Scan Cyclic Voltammetry

Voltammetry was performed as previously described [80,81]. Briefly, mice underwent rapid decapitation at 8–10 weeks of age and the right hemisphere was isolated. The right hemisphere was immersed in oxygenated sucrose aCSF solution (193 mM sucrose, 11 mM d-glucose, 1.2 mM CaCl_2_·2H_2_O, 4.5 mM KCl, 25 mM NaHCO_3_, 20.5 mM NaCl, 1.2 mM NaH_2_PO_4_, 2.6 mM MgCl_2_) for 30 s prior to sectioning at 300 µm using a Leica vibratome. Slices were incubated in oxygenated HEPES (4-(2-hydroxyethyl)-1-piperazineethanesulfonic acid) aCSF solution (19.7 mM HEPES, 11 mM d-glucose, 2.4 mM CaCl_2_·2H_2_O, 25 mM NaHCO_3_, 126.4 mM NaCl, 2.5 mM KCl, 1.2 mM NaH_2_PO_4_ monobasic, 2.6 mM MgCl_2_) for at least 30 min prior to testing. Five recordings were taken from five different sites along a single unilateral dorsal striatal slice for each animal. There was a 5-min interval between each 2.31 V stimulation. TarHeel CV Version 4.41 software (University of North Carolina, Durham, NC, USA) and a custom potentiostat (UEI, UNC Electronics Shop, Durham, NC, USA) were utilized for application of waveforms, stimulus, and current monitoring. To detect dopamine, a waveform of a −0.4 V holding potential versus an Ag/Ag Cl (In Vivo Metric, Healdsburg, CA, USA) reference electrode was used, with an applied voltage ramp from −0.4 V to 1.0 V and back at a rate of 600 V/s at 60 Hz. The maximal release was averaged for each striatal slice and carbon-fiber recording microelectrodes were calibrated with dopamine standards. Dopamine uptake was parameterized via tau, a time constant representing the amount of time necessary to return to 2/3 of baseline current after stimulation. Tau is derived from an exponential curve fitted to the dopamine current trace via a least squares constrained exponential fit algorithm that has previously been described and determined to be most accurate for measuring dopamine uptake compared to other kinetic parameters measured via fast-scan cyclic voltammetry [82]. Demon Voltammetry Version 110713 software (University of North Carolina, Durham, NC, USA) [82] was used to calculate kinetic constants describing release and uptake of dopamine, via nonlinear logistic regression.

### 2.8. Statistical Analyses

Statistical analyses were conducted in GraphPad Prism Version 8 (San Diego, CA, USA) unless otherwise indicated. A *t*-test was used to compare the serum corticosterone levels of female dams exposed to either deltamethrin or the vehicle. All other analyses investigated continuous outcomes in the offspring and utilized a one-way ANOVA because treatment had four levels: vehicle/vehicle, deltamethrin/vehicle, CORT/vehicle, and deltamethrin/CORT. We stratified by sex instead of using a two-way ANOVA because one male and one female offspring were utilized from each litter. These samples would thus violate the independent sample assumption because the litter was considered the smallest independent statistical unit. Additionally, one male and one female offspring were utilized from each litter for each experiment. Not all litters contained at least four males and four females and therefore there were differences in the number of subjects in some groups. However, the exposures themselves did not significantly affect body weight gain and survival (data not shown). A significance level of 0.05 was used throughout.

## 3. Results

### 3.1. CORT Exposure Decreases Serum CORT Independent of Deltamethrin Exposure 

**Figure 2:**
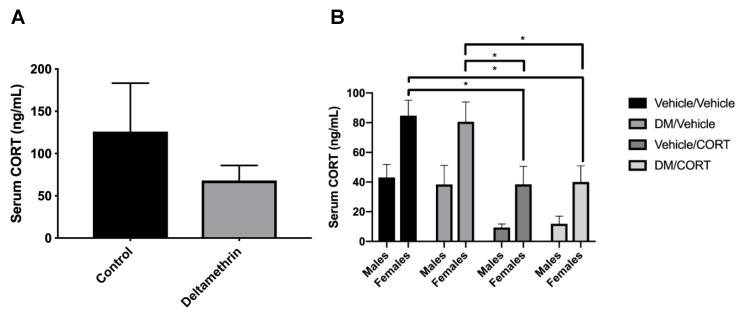
Exposure to oral CORT decreased endogenous CORT production independent of deltamethrin exposure in male and female offspring, and there was no significant difference in endogenous CORT production of female dams exposed to deltamethrin during gestation. We tested endogenous CORT levels via an ELISA assay to assess (**A**) whether deltamethrin exposure affects circulating corticosterone in the pregnant dams, which could subsequently affect in utero CORT levels observed by their offspring; and (**B**) whether CORT exposure affects circulating CORT in male and female offspring exposed to deltamethrin, oral CORT, or both. Exposure group differences were assessed via a two-tailed *t*-test for the exposed versus unexposed female dams (A) and via one-way ANOVA with Tukey’s post-hoc test, stratified by sex, for the male and female offspring (B). *N* = 7–8 in (A), *N* = 5–7 in (B), and error bars represent SEM. * *p* < 0.05.

To test whether deltamethrin exposure itself would affect circulating CORT levels in pregnant dams and potentially affect results observed in deltamethrin and CORT-exposed offspring, we assessed serum CORT levels via ELISA. We did not observe statistically significant differences in serum CORT of control and deltamethrin-exposed dams (125.90 ng/mL ± 57.38 vs. 68.02 ng/mL ± 17.87, *p* = 0.33) (Figure 2). We also did not observe significant alterations in serum CORT of deltamethrin-only exposed male and female offspring compared to the vehicle/vehicle group. However, we did observe significantly decreased serum CORT levels in the CORT-only and deltamethrin/CORT groups in females offspring compared to the vehicle/vehicle group (*p* = 0.01 and *p* = 0.01, respectively), indicating that the CORT exposure itself likely influenced circulating CORT levels independent of deltamethrin exposure in females. In addition, there was a significant overall effect of sex on serum CORT levels (*p* < 0.0001), illustrating sex-specific differences in circulating CORT levels. 

### 3.2. Sex-Specific Alterations in the Expression of Key Dopaminergic Genes

While we have previously studied alterations in the expression of key dopaminergic genes in the midbrain in response to dieldrin and heptachlor exposure, we had not yet investigated changes that occur in response to pyrethroid insecticide exposure [83,84]. Here, we sought to assess whether the expression of *Pitx3* and *Nurr1* transcription factors involved in development of the dopaminergic phenotype, as well as downstream dopaminergic targets *Th*, *Comt*, *Dat1*, and *Vmat2*, were altered in the midbrains of offspring exposed to deltamethrin, CORT, or both during neurodevelopment. We also assessed whether the expression of *Nr3c1*, the glucocorticoid receptor, would change in response to the aforementioned exposures. 

**Figure 3:**
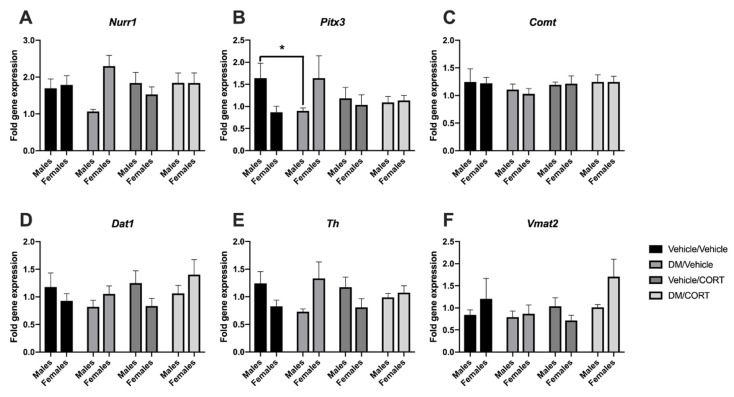
Midbrain *Pitx3* expression was significantly decreased in males exposed to deltamethrin/vehicle. Gene expression was assessed in the midbrain of male and female offspring exposed to one of four treatment groups: vehicle/vehicle, deltamethrin (DM)/vehicle, corticosterone (CORT)/vehicle, and DM/CORT. Midbrains were collected at 8–10 weeks of age via rapid decapitation and RNA was extracted. Gene expression was measured via Taqman assays and data was expressed as 2^ΔΔCt^ analysis of qPCR data and compared to expression in the vehicle/vehicle group. Exposure group differences were assessed via one-way ANOVA with Tukey’s post-hoc test and stratified by sex. * *p* < 0.05, *N* = 5–8, and error bars represent SEM.

*Pitx3* gene expression was significantly decreased in deltamethrin-only exposed males when compared to vehicle/vehicle exposed males (1.64 ± 0.34 vs. 0.90 ± 0.07, *p* = 0.04), but not deltamethrin-only exposed females (0.87 ± 0.13 vs. 1.6 ± 0.51, *p* = 0.29) (Figure 3B). There was also no significant difference in *Pitx3* expression in CORT-only and deltamethrin/CORT exposed males, suggesting that CORT exposure may mediate *Pitx3* expression when combined with deltamethrin exposure. Similar expression effects were observed for *Nurr1* transcription factor expression (Figure 3A), as well as for *Dat1* and *Th* (Figure 3D,E), though none of these effects reached statistical significance at α = 0.05. Interestingly, gene expression of *Pitx3*, *Nurr1*, *Dat1*, and *Th* trended upwards for females exposed to deltamethrin, opposite the trends observed in males, with an intermediate effect in the deltamethrin/CORT-exposed females (Figure 3). We did not see significant effects of deltamethrin and CORT exposure on mRNA expression of *Vmat2*, *Nr3c1*, or *Comt* in the midbrain (Figure 3).

### 3.3. TH and VMAT2 Expression Were Significantly Decreased in Striatum of Females Exposed to Deltamethrin/CORT, and TH Expression Was Significantly Decreased in the Frontal Cortex of Males Exposed to Deltamethrin

**Figure 4:**
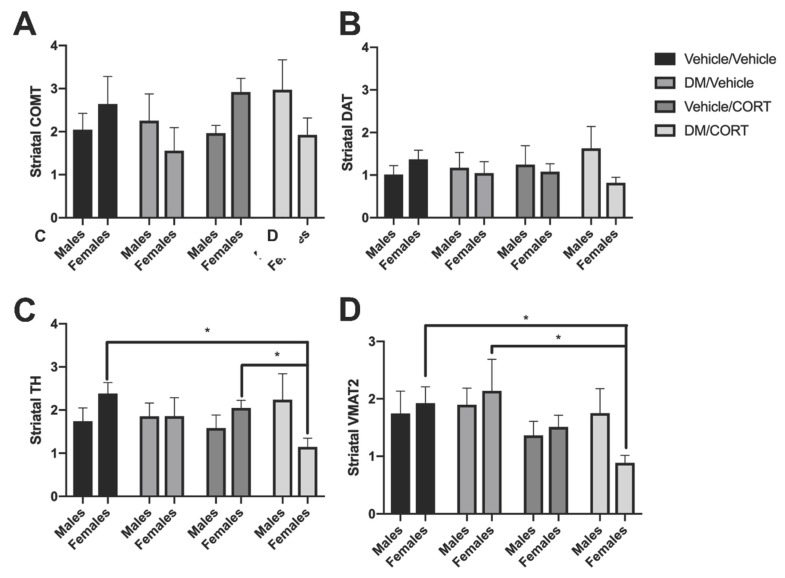
Striatal TH and VMAT2 expression was significantly decreased in female offspring exposed to deltamethrin (DM) and corticosterone (CORT). COMT (**A**), DAT (**B**), TH (**C**), and VMAT2 (**D**) protein expression in the striatum was assessed in male and female offspring exposed to one of four treatment groups. Striata were collected at 8–10 weeks of age. Protein expression is expressed as adjusted units (AU) assessed via densitometry analysis and normalized to actin. Exposure group differences were evaluated via one-way ANOVA with Tukey’s post-hoc test and stratified by sex. * *p* < 0.05, *N* = 5–7, and error bars represent SEM.

Previous studies have observed alterations in key dopaminergic proteins in response to various insecticide and chronic stress exposures in the striatum, a highly dopaminergic brain region [41,49,60,83,84,85,86,87,88]. We evaluated whether combined deltamethrin/CORT exposure would lead to additive effects on dopaminergic protein expression in the striatum via immunoblotting. In striata of females exposed to both deltamethrin and CORT, TH expression was significantly decreased compared to vehicle/vehicle control females (2.23 ± 0.24 vs. 1.15 ± 0.20, *p* = 0.01) (Figure 4C). There was a significant difference in TH expression between CORT-only exposed females and deltamethrin/CORT-exposed females, suggesting that dual exposure with deltamethrin may mediate the effect in females (2.47 ± 0.47 vs. 1.15 ± 0.45, *p* = 0.02) (Figure 4C). In addition, there was also a significant decrease in VMAT2 expression in the striata of deltamethrin/CORT-exposed females (1.92 ± 0.28 vs. 0.89 ± 0.13, *p* = 0.045) (Figure 4D). Furthermore, there was also a significant difference in VMAT2 expression between deltamethrin-only exposed females and deltamethrin-CORT-exposed females, suggesting that dual exposure with CORT mediates the effect on VMAT2 expression (2.14 ± 0.55 vs. 0.89 ± 0.13, *p* = 0.02) (Figure 4D). The effects on TH and VMAT2 expression were not observed in striata of males exposed to deltamethrin/CORT. There were no significant alterations in COMT and DAT expression in the striatum of males and females exposed to deltamethrin-only, CORT-only, and deltamethrin/CORT (Figure 4A,B). Additionally, we did not observe any changes in expression of NR3C1, which encodes the glucocorticoid receptor, in any of the exposure groups in males or females (data not shown).

**Figure 5:**
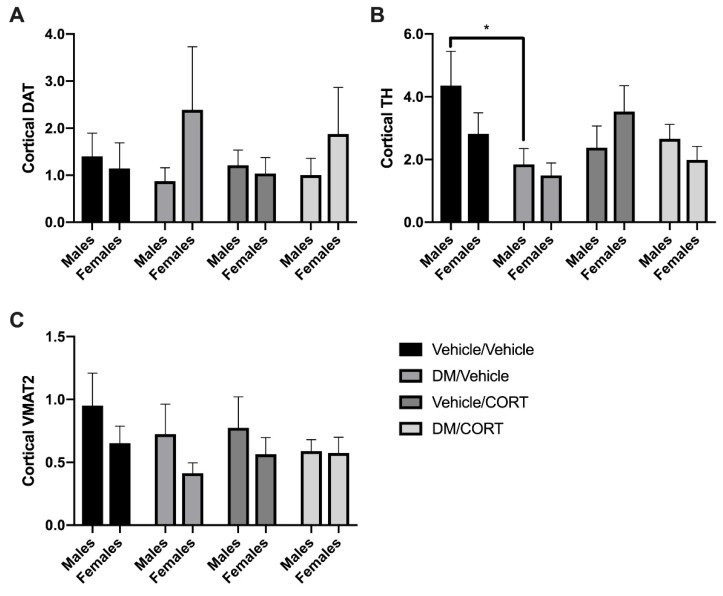
Cortical TH expression was significantly decreased in male offspring exposed to deltamethrin (DM) only. DAT (**A**), TH (**B**), and VMAT2 (**C**) protein expression in the frontal cortex of male and female offspring exposed to one of four treatment groups was assessed. Cortices were collected at 8-10 weeks of age. Protein expression is expressed as adjusted units (AU) assessed via densitometry analysis and normalized to actin. Exposure group differences were evaluated via one-way ANOVA with Tukey’s post-hoc analysis, stratified by sex. * *p* < 0.05, *N* = 5–7, and error bars represent SEM.

Next, we measured whether expression of key dopaminergic proteins change in response to deltamethrin and CORT exposure in the frontal cortex, another brain region that receives dopaminergic projections from the midbrain and has been associated with ADHD pathogenesis. There was a significant decrease in TH expression in the frontal cortex of deltamethrin-only exposed males compared to vehicle/vehicle controls (4.350 ± 1.098 vs. 2.380 ± 0.6870, *p* = 0.0340) (Figure 5C), but no significant difference in TH expression in dually-exposed males, suggesting that CORT may mediate the effect of deltamethrin on TH-expression. There is no significant change in expression of VMAT2, DAT, and TH of males and females exposed to deltamethrin-only, CORT-only, and deltamethrin/CORT when compared to the vehicle/vehicle control groups (Figure 5A,B). 

### 3.4. Deltamethrin Exposure Significantly Slowed Striatal Dopamine Uptake Rate in Males

**Figure 6:**
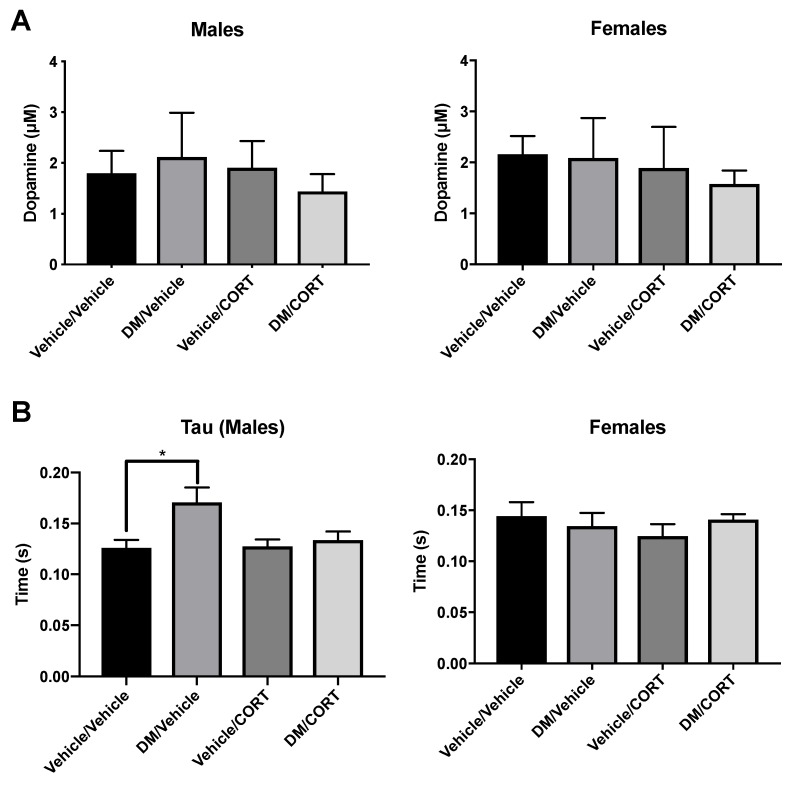
Significant impairment of dopamine uptake occurred in male offspring to deltamethrin (DM) only, with no significant change in peak dopamine release. Ex vivo electrochemical measurement of dopamine release and uptake via fast-scan cyclic voltammetry was measured in male and female offspring exposed to one of four treatment groups. In (**A**), peak dopamine release was measured in the dorsal striatum and averaged over all stimulation sites per animal. DM, corticosterone (CORT), or DM/CORT exposure did not significantly change peak dopamine release in male or female offspring. In (**B**), tau, a kinetic time constant, was used to characterize dopamine uptake into the synapse and tau values were averaged over all stimulation sites per animal. Deltamethrin exposure in males significantly increased tau, which represents a slowed and impaired dopamine uptake into the synapse. Exposure group differences were evaluated via one-way ANOVA with Tukey’s post-hoc analyses, stratified by sex. * *p* < 0.05, *N* = 5–7, and error bars represent SEM.

To investigate dopamine release and uptake dynamics ex vivo we employed fast-scan cyclic voltammetry as previously described [80,81,89]. This technique harnesses dopamine’s electrochemical properties and provides data on peak dopamine release in the striatum as well as dopamine uptake by DAT. Functionally, we also do not observe significant changes in peak dopamine release in any of the four exposure groups via voltammetry, in neither males nor females (Figure 6A). In contrast, we do observe a significant increase in the length of time necessary for dopamine uptake in striata of deltamethrin-only exposed males (Figure 6B). Dopamine uptake rate and, subsequently tau, were affected by changes to dopamine transporter function [82], suggesting that there may be more subtle changes in functionality but not overall DAT expression levels. There were no alterations in tau in dually-exposed deltamethrin/CORT males, suggesting that CORT may mediate the effect of deltamethrin on dopamine uptake dynamics (Figure 6B). These effects also appear to be sex-specific as there was neither a significant difference in dopamine release nor in tau in females in any of the exposure groups (Figure 6).

### 3.5. Males Exposed to Deltamethrin Display Increased Activity in Y Maze but Not Open Field

**Figure 7:**
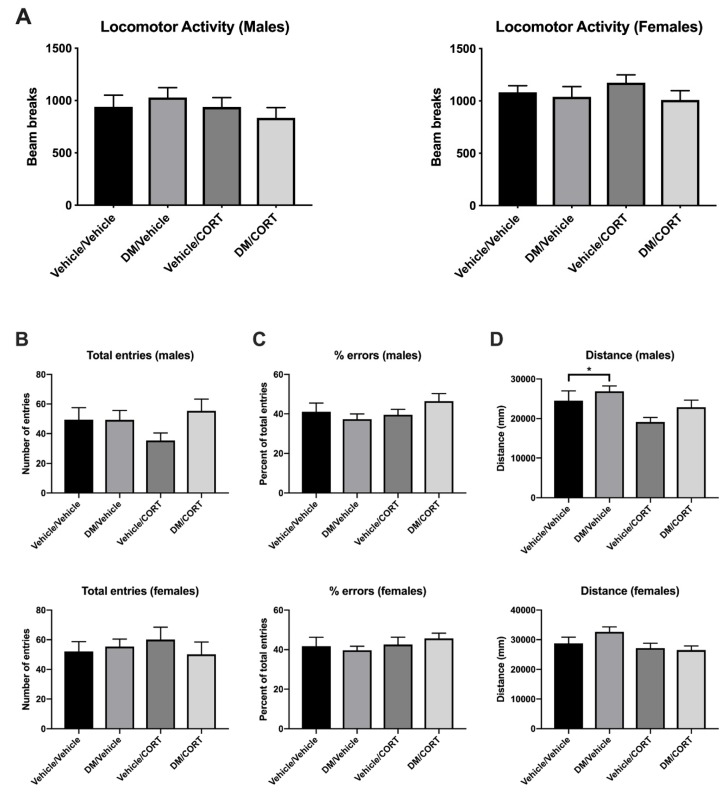
Males exposed to deltamethrin (DM) only exhibited increased distance traveled in the Y maze, but there were no significant differences in locomotor activity and Y maze attention performance in males and females exposed to DM, corticosterone (CORT), or both. Locomotor activity and Y maze assays were conducted in male and female offspring exposed to one of four treatment groups at 8 weeks of age. In (**A**) data are expressed as the average number of beam breaks in 60 min after a 30-min habituation period. In (**B**–**D**) mice were allowed to freely explore the Y maze for 8 min and arm entries were recorded to assess working memory, attention, and total distance traveled. Exposure group differences were assessed via one-way ANOVA with Tukey’s post-hoc analyses and stratified by sex. * *p* < 0.05, *N* = 7–9, and error bars represent SEM.

Given the alterations in mRNA expression, protein expression, and dopamine function, we next investigated whether these changes would result in ADHD-like behaviors. Hyperactivity is a cardinal feature of ADHD symptomology [2]. Genetic models of ADHD, as well as animal exposure models of environmental ADHD susceptibility, have tested locomotor activity through various methods [41,44,90,91,92,93]. We assessed locomotor activity over a one-hour period after mice were allowed to habituate to an open field box. We did not observe significant differences in the number of beam breaks in males or females in any of the exposure groups (*p* = 0.6199 for males, *p* = 0.5471 for females) (Figure 7A). There was also no significant difference in locomotor behavior during the habituation period (data not shown). 

There was no significant difference between exposure groups or sexes in alternation behavior or in the percentage of errors, suggesting that we did not detect alterations in working memory or attention in this assay (Figure 7B,C). However, males exposed to deltamethrin traveled a significantly longer distance in the Y-maze in 8 min compared to vehicle/vehicle control males (2452.9 cm ± 245.4 vs. 2690.8 cm ± 134.7, *p* = 0.0368), as well as when compared to males exposed to CORT (2452.9 cm ± 245.4 vs. 1915.0 cm ± 113.2, *p* = 0.0050) (Figure 7D). Thus, while mice did not display hyperactivity in the locomotor activity assay, they do display increased activity in the Y maze assay. This effect appears to be sex-specific, as there was no significant difference in distance traveled among the female exposure groups. Males dually exposed to deltamethrin and CORT did not travel an increased distance compared to the vehicle/vehicle control group (1915.0 cm ± 113.2 vs. 2284.3 cm ± 179.9 cm vs., *p* = 0.5109), suggesting that CORT exposure may mediate the hyperactivity effects of deltamethrin in the Y maze test. 

### 3.6. No Difference in Impulsivity as Measured Via Marble Burying in Animals Exposed to Deltamethrin and CORT

**Figure 8:**
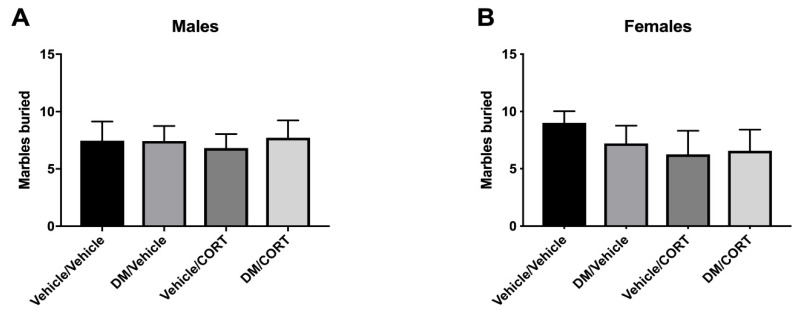
There was no significant difference in impulsivity, as measured by marble burying, in male (**A**) and female (**B**) offspring exposed to one of four treatment groups. Twenty marbles were placed in a 4 × 5 pattern on top of clean bedding. Offspring were tested at 8 weeks of age and allowed to roam freely for 30 min total. The number of marbles buried at least 50%, as scored by two independent scorers, is reported. *N* = 7–9, differences between exposure groups were assessed via one-way ANOVA with Tukey’s post-hoc analyses and were stratified by sex. Alpha level of 0.05 was used and error bars represent SEM.

Marble burying behavior has been used previously to test impulsivity in genetic models of ADHD predisposition [80]. While vehicle/vehicle control mice display expected burying behaviors, we did not observe significant differences in the absolute number of marbles buried at least 50% after 30 min in male or females in any of the exposure groups (*p* = 0.9754 for males, *p* = 0.6903 for females) (Figure 8).

## 4. Discussion

The etiology and pathogenesis of ADHD are still unclear, but ADHD continues to present a substantial public health problem and there is a need for additional research probing ADHD-associated neurologic pathways and sources of pathology. The existing data on ADHD suggest that alterations in dopaminergic signaling due to pyrethroid insecticide exposure influences ADHD pathogenesis and are important for studying ADHD risk [41,51]. Chronic stress exposure also alters the dopamine system and could further potentiate the risk of ADHD [60,87,94,95,96]. Given the independent effects of pyrethroid insecticides and chronic stress on the dopamine system, we hypothesized that combined pyrethroid insecticide and stress hormone exposure during critical periods of neurodevelopment would additively alter protein expression and the function of key components of the dopamine system, thereby further increasing the risk of developing ADHD.

### 4.1. Summary of Findings

In our study, we exposed pregnant dams to deltamethrin and their offspring to CORT dissolved in drinking water until adulthood and then employed a suite of behavioral and molecular approaches to gain a more comprehensive understanding of alterations to the dopamine system in response to dual exposures. Although deltamethrin did not affect serum CORT levels of male and female offspring, exposure to CORT in drinking water led to a decrease in serum CORT in male and female offspring exposed and unexposed to deltamethrin. Additionally, male offspring exposed to deltamethrin demonstrated a reduction in the expression of the midbrain transcription factor, *Pitx3*. Although changes in mRNA expression for specific dopaminergic phenotypic markers were not observed, a gender- and region-specific effect of TH and VMAT2 protein expression was detected. The functional consequences of these alterations manifested in a reduction in dopamine uptake in the striatum of male offspring exposed to deltamethrin, which corresponded with increased locomotor activity in the Y maze for these animals. 

### 4.2. Glucocorticoid Signaling and DM/CORT Exposure

No studies have previously examined the effects of developmental pyrethroid insecticide exposure on endogenous CORT production. Thus, our findings provide some novel insight into the effects of developmental exposure to deltamethrin on the hypothalamic–pituitary–adrenal (HPA) axis and CORT levels in offspring. While deltamethrin did not impact CORT levels at the time points we evaluated, we cannot rule out the possibility that assessment at earlier ages would have shown an alteration in CORT that did not persist as they aged and deltamethrin exposure was ceased. In contrast, a few studies have investigated the effects of oral CORT administration on serum CORT production. One study previously reported that adult male mice exposed to 25 μg/mL CORT in drinking water had significantly decreased serum CORT two weeks after exposure [97], but did not have significantly altered endogenous CORT production one month after exposure [77]. Additionally, females exposed to 35 μg/mL CORT for four weeks during adolescence show a significant decrease in plasma CORT levels after forced swim testing, suggesting a dampening effect on the normal stress response following exogenous CORT administration [98]. 

While these results support the reductions in CORT levels found in our study, these results run counterintuitive to our original hypothesis. Nonetheless, stimulation of the endogenous stress response generated by CORT exposure may provide some insight into the feedback mechanisms that mediate serum CORT levels in our model. In general, the stress response is mediated via actions of the HPA axis. Ordinarily, an acute stressor causes release of corticotropin-release factor (CRF) from the amygdala, subsequent adrenocorticotrophic hormone (ACTH) release from the pituitary, and glucocorticoid release from the adrenal glands. Glucocorticoids then inhibit the amygdala and pituitary gland via a negative feedback loop, leading to an overall reduction in CORT release [99,100]. Under circumstances of early life and chronic stress, HPA axis hyperactivity and impaired negative feedback are typically observed [99,100], though the type of stressor, sex, genetic predisposition, and environmental factors can all modulate the influence chronic stress has on maturation and function of the HPA axis and downstream CORT levels [101]. Thus, various inputs can impact the function of the HPA axis through alteration to the limbic nuclei as well as adrenal gland. Our results and those of others suggest that chronic oral CORT administration may impact the HPA axis centrally. A previous study showed that oral CORT given to rats during adolescence increases neuronal activity in the paraventricular nucleus of the hypothalamus and causes a blunted CORT response to restraint stress [102,103]. Additionally, chronic oral CORT was found to result in a reduction in plasma CORT, independent of damage or removal of the adrenal glands [104]. These findings support the central-acting effects of exogenous CORT in mediating serum CORT production and levels. Although deltamethrin did not result in a measurable change in CORT levels at the time points we measured, the impact of chronic CORT exposure suggests a more complex regulation of serum CORT levels, which could significantly affect the development of the dopamine system. 

### 4.3. Altered Midbrain RNA Expression

Through our novel neurodevelopmental combined exposure paradigm, we determined that midbrain expression of *Pitx3* was significantly decreased in adult males exposed to deltamethrin, with a similar trend in *Nurr1* expression. *Pitx3* and *Nurr1* are transcription factors with essential roles in the phenotypic development and maintenance of dopaminergic neurons in the midbrain [105,106,107]. *Pitx3* and *Nurr1* expression are normally induced by embryonic day (E) 11.5 in mice and are predominantly localized to the SNpc and VTA in the midbrain, where the densest population of dopamine neurons resides. Highlighting their function in dopamine neuron survival, genetic reduction of *Pitx3* or *Nurr1* in mice results in aberrant differentiation or loss of dopamine neurons in the midbrain and a concomitant loss of dopaminergic projections to the dorsal striatum [108,109,110,111,112,113,114]. While both transcription factors are critical in development of the dopamine system, *Nurr1* helps determine and maintain a neuron’s dopaminergic phenotype. Conversely, *Pitx3* does so only for a laterally-located subset of midbrain dopaminergic neurons in the SNpc [115]. Lateral subpopulations in the SNpc that express *Pitx3* are also more susceptible to the dopaminergic neurotoxicant, MPTP [116]. Perhaps, we detected *Pitx3* but not *Nurr1* alterations because Pitx3-expressing subpopulations are more sensitive to neurotoxic dopamine perturbations by deltamethrin than other subtypes of dopaminergic neurons found in this region. As far as we are aware, these are the first data evaluating the impact of developmental deltamethrin and CORT exposure on transcription factors associated with development of the mesencephalic dopamine region. 

Interestingly, although we observed changes in transcription factors that modulate *Dat1*, *Vmat2*, and *Th* expression, we did not find observable changes in overall expression of these genes in the adult midbrain. While we were able to measure alterations to *Pitx3*, it may be that these changes were not robust enough to elicit a significant change in mRNA expression of downstream genes. Additionally, alterations in gene expression of these dopaminergic components may be transient and do not persist into adulthood after the exposure to deltamethrin and CORT, making it difficult to capture these changes in our exposure paradigm.

### 4.4. Altered Striatal and Cortical Protein Expression

To our knowledge no one has assessed striatal COMT, DAT, TH, and VMAT2, nor has anyone assessed cortical DAT, TH, and VMAT2 after combined exposure to deltamethrin and CORT during neurodevelopment. Utilizing this paradigm, we found no explicit changes in these proteins with CORT exposure alone but did observe changes in striatal TH and VMAT2 following exposure to combined deltamethrin and CORT, while cortical TH was reduced after deltamethrin alone. The striatal expression of TH and VMAT2 in female offspring exposed to a combination of deltamethrin and CORT was significantly decreased, while expression of these proteins remained unchanged in male mice. Previous studies have observed increases in striatal DAT expression at 6 weeks of age following a neurodevelopmental exposure to 3 mg/kg deltamethrin [41]. We measured striatal DAT expression at 8–10 weeks and did not observe this increase, possibly because the effect of deltamethrin on striatal DAT expression does not persist into adulthood using this exposure paradigm. 

Previous studies that have investigated the dopaminergic effects of psychosocial stress provide a few possible explanations for the consequences of CORT in dopaminergic neurodevelopment in our study. Rodents exposed to various forms of prolonged behavioral psychosocial stress display decreased density and binding of DAT in the dorsal striatum [87], dorsolateral caudate putamen [60], and nucleus accumbens [60]. However, these results were observed in male tree shrews [87] and male rats [60] exposed to psychosocial stress in adulthood. Since we employed a neurodevelopmental exposure paradigm and male and female mice received oral CORT from weaning through adolescence, this differential stress exposure paradigm might explain why we did not observe any changes in DAT expression in the striatum and frontal cortex. Potentially, deltamethrin exposure from gestation through weaning increased the susceptibility of dopaminergic neurons in the striatum, and subsequent exposure to oral CORT from adolescence to adulthood then had additional neurotoxic effects that led to a measurable decrease in TH and VMAT2. This could help explain why we did not observe decreased striatal TH and VMAT2 in the deltamethrin-only and CORT-only groups. 

In contrast to our findings in the striatum, adult males exposed to deltamethrin-only during neurodevelopment showed significantly decreased TH expression in the cortex. This change in males is not present in the dually-exposed deltamethrin/CORT group. These findings suggest a differential impact of deltamethrin and CORT on the mesolimbic circuitry compared with the nigrostriatal circuitry. 

### 4.5. Functional Consequences of Deltamethrin and CORT Exposure

Next, we investigated whether the molecular changes we observed would incur functional consequences via ex vivo fast-scan cyclic voltammetry and a battery of behavioral assays. Via electrochemical analysis of dopamine release dynamics, we observed that males exposed to deltamethrin did not exhibit differences in peak dopamine release in the striatum, but males exposed to deltamethrin did show a significant impairment in dopamine uptake. In our electrochemical voltammetry studies, dopamine uptake was parameterized as the time constant tau. Tau describes the amount of time to return to 2/3 of baseline current [82,117] and is increased by administration of known pharmacologic dopamine uptake inhibitors, such as amantadine [118,119], as well as cocaine [119]. Our results were surprising, given the lack of effect of deltamethrin exposure on DAT expression in the striatum. These findings suggest that developmental exposure to deltamethrin can elicit a significant impairment in DAT function, that is independent of concomitant changes in DAT expression. 

DAT is an integral membrane protein that sequesters cytosolic dopamine into vesicles, thereby controlling dopaminergic signaling pre- and post-synaptically [120]. It acts as a symporter to bind two sodium ions, one chloride ion, and the dopamine substrate, moving between inward and outward facing states relative to the dopamine vesicle [121]. Genetic and pharmacologic manipulation studies of DAT illustrate that DAT function could be affected in many ways [122]. Previous studies show that the DAT transport function can be impacted by binding in different conformational states, for example. Cocaine and methylphenidate, a first-line ADHD medication, stabilize DAT’s outward-facing conformation [123], whereas other neuropsychiatric medications, such as bupropion and modafinil, stabilize the inward-facing conformation [124]. Conformation changes in DAT are critical for its proper function as a symporter, as it must move between these conformational states to properly transport dopamine. Perhaps, deltamethrin and CORT exposure abnormally stabilize or impair one of these formations and this leads to a slowed mechanism of dopamine uptake that is independent of changes in DAT expression or dopamine release. There are a few examples of functional *DAT1* mutations that confer changes in dopamine uptake, but not necessarily dopamine release or expression. One group transfected a human *DAT1* mutation associated with autism spectrum disorder, T365M, into cells and observed significantly lower maximal velocity of dopamine influx. However, neither the affinity of dopamine nor overall expression of DAT was affected [125]. In another in vitro study of two *DAT1* mutations found in an adult patient with comorbid Parkinson’s disease and ADHD, the DAT1 I312D mutation was associated with significantly lower maximal velocity of dopamine influx but no difference in overall DAT protein expression as well [126]. Thus, in alignment with the aforementioned studies, we did not observe significantly altered DAT expression or release in the striatum of males exposed to deltamethrin/CORT, but dopamine influx velocity was significantly decreased. Dual deltamethrin/CORT exposure seems to attenuate the effect of deltamethrin on dopamine uptake in the striata of adult males, suggesting that CORT exposure could still play a role in determining the rate of dopamine uptake. 

Given the observed changes in mRNA expression, protein expression, and DAT functionality, we then tested ADHD-like behaviors. Interestingly, males exposed to deltamethrin demonstrate hyperactive behavior in the Y maze, though no differences in attention or impulsivity were measured. It is possible that our behavioral endpoints were not sensitive enough to detect changes at this level, especially given the lack of alterations in DAT expression and peak dopamine release ex vivo.

## 5. Conclusions

In summary, we established a neurodevelopmental exposure paradigm of joint environmental factors that resulted in novel CORT reduction, RNA expression, striatal and cortical protein expression, and dopamine uptake rate findings. These findings contribute to our understanding of potential mechanisms of action of deltamethrin and oral CORT, both alone and in combination. Due to the dopaminergic effects previously described by our group and others after exposure to either deltamethrin alone or psychosocial stress or CORT alone, we hypothesized there would be synergistic effects in the dopamine system after developmental exposure to these in combination. Interestingly, we did not observe significant synergistic effects of the combined deltamethrin/CORT exposure on mRNA and protein expression of several key components within the dopamine system. Instead our data suggest that dual exposure to the major stress hormone CORT may actually dampen the effects of deltamethrin on the development of the dopamine system in males. Our study demonstrates a broad effect on the development and function of the dopamine circuit, which may be impacted by temporal aspects of deltamethrin and CORT exposure during neurodevelopment. The rationale for following up on these results is based on our understanding of the “multiple hit” hypothesis, which suggests environmental exposures render a biological system more vulnerable to subsequent environmental insults [127]. In the context of this study, while the initial exposures to deltamethrin or CORT may not manifest in an overt pathological effect on the dopamine circuit, future exposures to stress, deltamethrin, or other environmental factors could unmask more profound neurotoxicological endpoints. Aligned with this, our study highlights the importance of testing multiple environmental exposures in conjunction to better understand how combined exposures present in real-life can affect similar neurodevelopmental systems and could differentially affect particularly vulnerable pediatric populations. First-line treatment with methylphenidate, a stimulant, resolves symptoms of the three domains for the majority of children with ADHD, but treatment response is variable and use is associated with stunted growth [128], neural plasticity effects [129], and substance abuse [130]. Studies also indicate that children with ADHD report deficits in psychosocial well-being and family life [131]. Thus, while significant clinical and research advancements have been realized, ADHD continues to present a major public health burden. Further study of predisposing mechanisms and pathophysiology is therefore merited. 

## Supplementary Materials

The following are available online at https://www.mdpi.com/2305-6304/7/2/24/s1, Table S1: Taqman Gene Expression Assays.
