# Supplementary material for: A Neurodevelopmental Model of Combined Pyrethroid and Chronic Stress Exposure
Source: Toxics. 2019 May 2;7(2):24. doi: 10.3390/toxics7020024 (PMC6630986; doi:10.3390/toxics7020024)
Supplement: Supplementary file 1 [file toxics-07-00024-s001.pdf]

toxics

Supplementary Materials: A Neurodevelopmental Model of Combined Pyrethroid and Chronic Stress Exposure

Aimée I. Vester, Merry Chen, Carmen J. Marsit and W. Michael Caudle

Table S1. Taqman Gene Expression Assays.

Gene of Interest	Assay ID
β-actin	Mm02619580_g1
Dat1/Slc6a3	Mm00438388_m1
Vmat2/Slc18a2	Mm00553058_m1
Comt	Mm00514377_m1
Th	Mm00447557_m1
Nurr1/Nr4a2	Mm00443060_m1
Pitx3	Mm01194166_g1
Nr3c1	Mm00433832_m1
